# Setting foot in private spaces: extending the hepatitis C cascade of care to automatic needle/syringe dispensing machines, a mixed methods study

**DOI:** 10.1186/s12954-022-00640-6

**Published:** 2022-05-28

**Authors:** Heidi Coupland, Charles Henderson, Janice Pritchard-Jones, Shih-Chi Kao, Sinead Sheils, Regina Nagy, Martin O’Donnell, Paul S. Haber, Carolyn A. Day

**Affiliations:** 1 Edith Collins Centre (Translational Research in Alcohol, Drugs and Toxicology), Sydney Local Health District, Sydney, Australia; 2 Drug Health Services, Royal Prince Alfred Hospital, Sydney Local Health District, Sydney, Australia; 3 Specialty of Addiction Medicine, Faculty of Medicine and Health, Central Clinical School (C39), The University of Sydney, Sydney, NSW 2006 Australia; 4 AW Morrow Gastroenterology and Liver Centre, Royal Prince Alfred Hospital, Sydney Local Health District, Sydney, Australia; 5 New South Wales Users and AIDS Association, Surry Hills, Sydney, Australia; 6 HIV and Related Programs (HARP) Unit, Population Health, Sydney Local Health District, Sydney, Australia

**Keywords:** Automatic syringe dispensing machines, People who inject drugs, Hepatitis C, Engagement

## Abstract

**Background:**

Global commitment to achieving hepatitis C virus (HCV) elimination has enhanced efforts in improving access to direct-acting antiviral (DAA) treatments for people who inject drugs (PWID). Scale-up of efforts to engage hard-to-reach groups of PWID in HCV testing and treatment is crucial to success. Automatic needle/syringe dispensing machines (ADMs) have been used internationally to distribute sterile injecting equipment. ADMs are a unique harm reduction service, affording maximum anonymity to service users. This paper explores the feasibility and acceptability of extending the HCV cascade of care to sites where ADMs are located.

**Methods:**

The ADM users into Treatment (ADMiT) study was undertaken in a metropolitan region in Sydney, Australia. This mixed methods study involved analysis of closed-circuit television footage, ethnographic methods (fieldwork observation and in-depth interviews) and structured surveys. Researchers and peers conducted fieldwork and data collection over 10 weeks at one ADM site, including offering access to HCV testing and treatment.

**Results:**

Findings from 10 weeks of fieldwork observations, 70 survey participants and 15 interviews highlighted that there is scope for engaging with this population at the time they use the ADM, and enhanced linkage to HCV testing and treatment may be warranted. Most survey participants reported prior HCV testing, 61% in the last 12 months and 38% had received HCV treatment. However, fieldwork revealed that most people observed using the ADM were not willing to engage with the researchers. Field work data and interviews suggested that extending the HCV cascade of care to ADMs may encroach on what is a private space for many PWID, utilized specifically to avoid engagement.

**Discussion:**

Enhanced linkage to HCV testing and treatment for people who use ADMs may be warranted. However, data suggested that extending the HCV cascade of care to ADMs may encroach on what is a private space for many PWID, utilized specifically to avoid engagement. The current study raises important public health questions about the need to ensure interventions reflect the needs of affected communities, including their right to remain anonymous.

## Introduction

Automatic needle/syringe dispensing machines (ADMs) have been used to distribute sterile injecting equipment in many countries as part of needle and syringe programmes (NSPs). As a keystone of harm reduction, the primary aim of NSPs is to prevent blood-borne virus infection among people who inject drugs (PWID) [1, 2]. ADMs are a cost-effective public health strategy for enhancing NSP coverage, particularly outside business hours [3, 4], and are a highly acceptable and convenient service for PWID provided machines are kept stocked and in working order [5, 6]. Many PWID prefer ADMs to maintain anonymity and express concerns about stigma and discrimination and attracting police attention [7, 8].

The availability of highly efficacious direct-acting antiviral (DAA) treatments that cure hepatitis C virus (HCV) infection has resulted in considerable reconfigurations in the global policy focus in relation to HCV prevention. While increasing NSP coverage remains a priority [9], mathematical modelling suggests that DAA treatment as prevention can contribute to substantially curbing incident HCV infections and ultimately achieve global HCV elimination [10]. The World Health Organization reports that HCV elimination will require an estimated 90% reduction in new HCV infections and 80% of eligible people treated for HCV [11].

In Australia, a range of strategies for increasing HCV testing and treatment uptake by PWID have been implemented and over 70,000 Australians have been treated [12]. Identifying hidden populations who are not engaged with health services, and supporting their engagement in HCV testing and treatment, is fundamental to success [13]. Fixed site, mobile and outreach NSPs, traditionally conceived as harm reduction services, have been found to be acceptable sites for HCV testing for PWID [14, 15]. However, the potential role of ADMs in supporting linkage to the HCV care cascade is yet to be explored.

People who use ADMs may represent a hidden group with limited engagement in HCV-related care. International literature suggests that ADMs can increase the availability of sterile injecting equipment for PWID who may not be engaged with NSPs or drug treatment services [6, 16]. Several studies have shown that some people who use ADMs differ from those who access face-to-face NSPs or pharmacies for injecting equipment, reporting that primary ADM users were younger, had shorter durations of injecting, injected less frequently, were less likely to report risky injecting practices or to have received opioid agonist treatment (OAT) [5, 7, 17, 18]. However, two recent Australian studies suggested that when ADMs were located adjacent to face-to-face NSPs and near pharmacies, the ADM was primarily used by clients of face-to-face services who used the ADM outside business hours [19, 20].

In regard to HCV prevention and testing, some studies report that people who use ADMs report less syringe sharing [16] and do not differ from those who primarily use face-to-face NSPs in terms of HCV risk practices [8, 20, 21]. However, uptake of HCV testing and particularly treatment among people who use ADMs remains unclear, including potential gender differences. Research suggests women may experience gender-specific barriers to HCV care [22, 23] and may be reluctant to access face-to-face drug health services because of stigma [24, 25] and concerns about child protection [26]. ADMs are a unique addition to the constellation of services offered by NSPs, affording maximum anonymity to service users. However, whether the HCV cascade of care should be extended to sites where ADMs are located, is unknown.

As part of ongoing, concerted efforts to engage hard-to-reach groups in HCV care, the ADM users into Treatment (ADMiT) study was undertaken in 2020, in a metropolitan region in one of Australia’s largest cities. ADMiT was a mixed-methods study conducted at one ADM site, aimed at determining who used the ADM and whether they were an important group to target for linkage to HCV care. Here we report selected findings to examine the characteristics of the population using the ADM and explore the feasibility and acceptability of engaging PWID in HCV testing at the time they used the ADM.

## Materials and methods

### Setting

The ADM is located at a large inner-city teaching hospital and distributes more than one-quarter of the half-million needles and syringes distributed districtwide through five ADMs each year. Injecting equipment is dispensed free-of-charge in “fit-packs” or black plastic boxes containing six needles and syringes, spoons, cotton wool, sterile water and swabs.

### Design

**Fig. 1:**
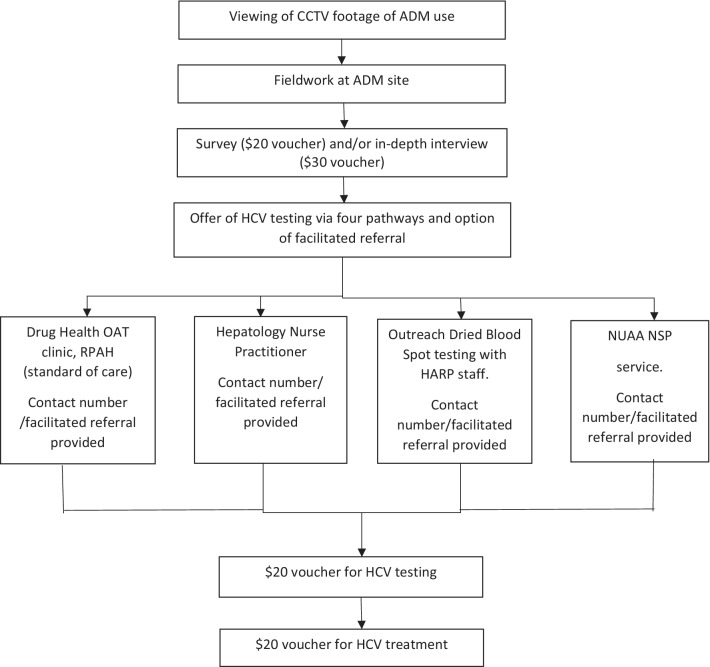
Overview of methodology

Ethnographic methods (fieldwork observation and in-depth interviews) and structured brief surveys were used in this mixed methods study (Fig. 1). Initially, two weeks of closed-circuit television (CCTV) footage of people using the ADM was viewed by a researcher (HC) to determine periods of heaviest ADM use and inform the fieldwork schedule. Fieldwork was conducted in blocks over a 10-week period. Observations were made on each day of the week, between the hours of 0.800 and 24.00, and 24.00 and 3.00 on weekends, to observe people using the ADM, recruit survey and interview participants and offer HCV testing. The fieldwork team included a researcher (HC) and up to two peer workers who were men and women aged in their 40 s and 50 s. Fieldwork was conducted within 10 m of the ADM, ensuring people could freely access the ADM without engaging, if preferred. A survey recruitment flyer was placed on the ADM or potential participants could approach researchers directly when they used the ADM. Fieldnotes were recorded by the researcher, who was an experienced ethnographic researcher (HC), and for each observed ADM occasion of use, the following were recorded: gender, approximate age, number in group, transport used, number of fit-packs obtained, and whether the survey was completed.

The survey was developed in consultation with the Project Steering Committee and all people observed using the ADM were eligible to participate. Participants provided verbal consent and received a $20 grocery voucher for participation and an offer of HCV testing via four possible pathways: an adjacent drug health service providing OAT; a nurse practitioner; a peer-operated NSP; or Outreach Dried Blood Spot (DBS) testing at a sexual health service. Interested participants were offered contact details for the service of their choice or facilitated referral, where the researcher contacted the service or made a referral on the participant’s behalf, using a pseudonym. Participants who self-identified as study participants when they accessed the service received an additional $20 voucher for HCV testing and for initiating treatment. At the end of the study, testing services provided the research team with de-identified information regarding uptake of HCV testing and treatment by those who identified as participants and accessed the service.

Some participants were invited to participate in a semi-structured in-depth interview at a time convenient for them. While all PWID who used the ADM were eligible, a purposive sampling frame was adopted, to recruit people of varied gender, age, drug use patterns and contact with health services. Digitally-recorded interviews were conducted by the researcher (HC) and transcribed verbatim. Topics explored included drug use patterns, patterns of ADM use, engagement with health services, HCV risk, prevention, testing and treatment. Participants gave written consent and received a $30 voucher for participating. Ethics approval was granted by the Human Research Ethics Committee (2020/ETH01023).

### Data analysis

Data from fieldwork observations of ADM use and survey data were entered into SPSS. Survey data frequencies were reported for categorical data and medians and ranges for continuous data. Gender differences for variables of interest were determined using chi-square for tests for categorical data and, where applicable, Fisher’s exact test. For continuous data, the Mann–Whitney-U Statistic was calculated to compare medians. Significance was set at *p* < 0.05 for all tests. De-identified HCV testing and treatment uptake data were obtained from services accessed by participants, and collated.

The lead researcher (HC) conducted the qualitative analyses. Fieldnotes were read and re-read, and main topics were coded by recording notes in the margins and then collating data on emerging themes. Interview data were entered into NVivo and analysed using a constant comparative approach. Initial parent codes were broadly based on the topic guide for interviews. Additional parent and child nodes were incorporated into the coding framework as new information emerged in the data. Key themes were then identified.

## Results

Quantitative and qualitative data highlighted the characteristics of the people who used the ADM, factors influencing engagement with people at the time they came to use the ADM, and uptake of HCV testing and treatment.

1. *Who uses the ADM?*

### Fieldwork observations of occasions of use

Non-consecutive sessions of fieldwork (*n* = 27) were conducted over 10-weeks from September to November 2020. There were 201 occasions of use observed, of which 33 (16%) were repeat uses and 16 (8%) were by people who had already completed the survey. Of the 201 occasions of use observed, highest use was on Saturdays (21%) and between 16.00 and 19.59 h (31%; Table 1).

**Table 1:** Fieldwork-observed occasions of ADM use by gender

	Total (%) *n* = 201	Females (%) *n* = 50	Males (%) *n* = 149	*p*-value
Day of week^a^				
Monday	21 (10)	5 (10)	16 (11)	
Tuesday	21 (10)	6 (12)	15 (10)	
Wednesday	31 (15)	9 (18)	22 (15)	
Thursday	22 (11)	7 (14)	15 (10)	
Friday	40 (20)	6 (12)	33 (21)	
Saturday	42 (21)	10 (20)	31 (21)	
Sunday	24 (12)	7 (14)	17 (11)	.794
Time of day^b^				
2400–0300	11 (5)	4 (8)	7 (5)	
0301–0759	0 (0)	0 (0)	0 (0)	
0800–1159	38 (19)	8 (16)	30 (20)	
1200–1559	45 (22)	11 (22)	34 (23)	
1600–1959	63 (31)	18 (36)	44 (30)	
2000–2359	44 (22)	9 (18)	34 (23)	.738
Age categories (years)				
20 s	11 (5)	6 (12)	5 (3)	
30 s	70 (35)	15 (30)	55 (37)	
40 s	84 (42)	20 (40)	62 (42)	
50 s+	36 (18)	9 (18)	27 (18)	.228
Number in group				
Alone	150 (75)	30 (60)	120 (81)	
≥ 2	51 (25)	20 (40)	29 (20)	.004
Transport				
On foot	129 (64)	32 (64)	97 (65)	
Car	59 (29)	15 (30)	42 (28)	
Other^c^	13 (6)	3 (6)	10 (7)	.961
Fit-packs obtained/use				
1–2	171 (85)	36 (72)	133 (89)	
≥ 3	30 (15)	14 (28)	16 (11)	.003
Disposal				
Yes	3 (1)	2 (4)	1 (1)	
No	198 (99)	48 (96)	148 (99)	.095

^a^Includes duplicate fieldwork sessions on Wednesday (*n* = 2) and Friday (*n* = 2)

^b^Fieldwork occasions of use for 2400 -259 for Saturday and Sunday only; No fieldwork occasions of use observed from 0300- 0759 h

^c^Other transport include motorbike or bicycle (*n* = 9) and scooter or skateboard (*n* = 3)

Fieldwork observations suggested that a highly diverse population used the ADM including people aged 20–70 years and from varied cultural backgrounds. Some people appeared to be experiencing homelessness and arrived at the ADM carrying bags full of clothing and sleeping bags. Some people showed signs of acute mental health problems.

Most occasions of use were by people who appeared to be male (74%) and people aged over 40-years (60%). Only two occasions of use were by people who appeared to be transgender, so these were excluded from analysis of gender only. Three-quarters of occasions of use were by unaccompanied people (75%), and most people obtained one or two fit-packs (85%; range 1–20). Although only one-quarter of the occasions of use were by females (Table 1), compared to males, and females were significantly more likely to be accompanied by one or two others (40% vs 20%; *p* 0.004) and to obtain more (> 3) fit-packs (28% vs 11%; *p* 0.003; Table 1).

During informal interactions, some people described limited contact with health services at that time, particularly among those who injected methamphetamine and, occasionally, mothers and people injecting heroin who had never been in OAT, or had recently stopped. The ADM was considered a highly valued service by all participants.

### Survey data

Thirty-five percent of occasions of use during fieldwork resulted in survey participation. Of seventy participants completing the brief survey, 28 (40%) identified as female, 41 (59%) as male and one (1%) as non-binary (Table 2). Participants (*n* = 70) reported a median age of 42 years (range 21–67 years) and most (83%) were local district residents. Three participants reported they did not inject and were obtaining fit-packs for others. Of the 67 participants who did inject, most (53%) reported less than daily injecting. Due to small sample sizes, limited gender analyses could be conducted. Female participants were significantly more likely than males to report injecting daily or more (59% vs 32%; *p* = 0.026). Methamphetamine was the most commonly reported last drug injected (58%), with no statistically discernible differences between males and females (Table 2).

**Table 2:** Survey participants’ demographic characteristics and drug use patterns

	Total (%) *n* = 70	Female (%) *n* = 28	Male (%) *n* = 41	*p*-value
Age (years)				
20 s	9 (13)	5 (18)	4 (10)	
30 s	19 (27)	7 (25)	11 (27)	
40 s	25 (36)	10 (36)	15 (37)	
50 s+	17 (24)	6 (21)	11 (27)	0.822
Median (range)	42 (21–67)	42 (21–67)	43 (25–61)	0.625^a^
Residential Postcode				
Local health district	58 (83)	26 (93)	31 (77)	
Other^b^	12 (17)	2 (7)	9 (23)	0.108
Last drug injected^c^				
Heroin	21 (31)	11 (41)	9 (23)	
Methamphetamine	39 (58)	15 (56)	24 (62)	
Other^c^	7 (10)	1 (4)	6 (15)	0.150
Frequency of injecting^c^				
Daily or more	29 (41)	16 (59)	12 (32)	
< Daily	37 (53)	11 (41)	26 (68)	0.026

^a^Mann–Whitney U = 614

^b^Other: Other health districts (*n* = 11); Missing (*n* = 1)

^c^Doesn’t inject (*n* = 3) excluded from the analysis; ^c^Other: Methadone (*n* = 1); fentanyl (*n* = 1); ketamine (*n* = 1); human growth hormone (*n* = 1); unspecified (*n* = 3)

Of the 69 participants who answered survey questions about HCV testing and treatment, 63 (91%) reported prior HCV testing (Table 3) and 33 (61%) reported being tested in the previous 12-months. Fifty-three participants provided the place last tested; prison (28%) and OAT clinic (25%) were the most common. Twenty-six participants (38%) reported prior HCV treatment. For most, treatment was more than 12-months prior. Prison and OAT clinics were the most common treatment locations (Table 3).

**Table 3:** Time and place of prior HCV testing and treatment

HCV testing	*n* = 54 (%)
Gender	
Female	21 (39)
Male	32 (59)
Non-binary	1 (2)
When last test?	
< 12 months ago	33 (61)
12 months+	21 (39)
Where last HCV test?^a^	
Prison	15 (28)
OAT clinic	13 (25)
General practitioner	8 (15)
Targeted primary health care	8 (15)
Sexual health clinic	4 (8)
Other^b^	5 (9)

HCV treatment	*n* = 23^c^ (%)
When last treated?	
Currently	3 (13)
< 12 months prior	3 (13)
1–5 years prior	10 (43)
5+ years (interferon)	7 (30)
Where last treated?^d^	
Prison	9 (45)
OAT clinic	6 (30)
Other^e^	5 (25)

^a^Sample providing testing place information *n* = 53

^b^Other: NSP (*n* = 2); Liver clinic (*n* = 1); Housing service (*n* = 2)

^c^Sample providing treatment information *n* = 23

^d^Sample providing treatment place information *n* = 20

^e^Other includes: Targeted primary health care (*n* = 2); General practitioner (*n* = 1); Sexual health clinic (*n* = 1); Housing service (*n* = 1)

Forty-six participants (66%) reported using the ADM weekly or less. Of the 69 survey participants who reported the number of fit-packs obtained, half (53%) obtained one or two fit-packs per week. Most participants (60%) obtained fit-packs for themselves and at least one other person (range 1–20). Reasons for using the ADM were convenience (84%), equipment being free-of-charge (44%) and anonymity (21%).

Of the 57 survey participants reporting accessing services in relation to their drug use, 30 (53%) had ever been in OAT, 19 (36%) had used a targeted primary health care service and 12 (21%) had used face-to-face NSPs. Only 7% reported accessing only ADMs and pharmacies.

2. *Factors influencing engagement*

Fieldnotes and in-depth interview data highlighted that people’s willingness to engage with the research team varied. Some people were very comfortable approaching the researchers and on several occasions, stayed talking with the research team for some time after using the ADM.

Vouchers ($20 and $30) provided for research participation, and as an incentive for HCV testing and treatment uptake, appeared to be an effective strategy supporting engagement. The involvement of peer workers also proved valuable. Sometimes peers were recognized because of their work at a well-known NSP. Peers’ lived experience could also reassure participants and facilitate rapport-building and honest responses to survey questions.

On several occasions, the expertise of peers regarding injecting practices was gratefully received by people using the ADM and assisted with building trust.

We saw Tracey (27 years old) at about 7pm. She’d just jumped off 80mg of methadone and was injecting methamphetamine and hadn’t slept for five days. However, she could still feel the effects of withdrawal. “I really need heroin” she said. “I’m so tired. But I like it. I don’t want to sleep. I hate sleeping”. She mentioned her arm was really hurting and the peer spoke to her, “Darl, you need to eat and drink something, even a banana”. The peer talked to her about injecting practices as she was having a lot of trouble finding a vein and kept “missing”. The syringe kept blocking because the drug mix (meth) contained congealing blood and this caused even more stress because she could see her money going to waste. She had tried squirting the mix back onto a spoon and diluting it and sucking it back up, but that didn’t fix the problem. And there was “blood everywhere”. (31/10/2020)

However, engagement beyond a brief exchange often proved challenging. Three factors raised questions about the acceptability of engaging with people at the time they used the ADM: embarrassment and anonymity; lack of time; and intruding on a private space.

### Embarrassment and anonymity: Who didn’t engage?

While only 21% of survey participants reported concerns about anonymity when using the ADM, interview data and fieldnotes suggested these concerns may have been more common for the broader population using the ADM, particularly among those who did not engage with the researchers.

Participants interviewed in-depth (*n* = 15) were aged between 27 and 67 years and eight were female. Methamphetmine injecting was common (11 reported it as the last drug injected), but most (*n* = 12) reported a history of injecting more than one drug; seven were in OAT. Four interviewees injected daily or more, all of whom were female. With one exception, participants reported prior HCV testing, but six had not been tested in the last 12 months. Six of those interviewed reported prior HCV treatment, all but one with DAAs.

Many interview participants, including those who had been in OAT or had accessed face-to-face drug health services, described using the ADM as quickly as possible to avoid being seen.

I just try to be as quick as possible and look around and try to avoid being seen by people. (Kate, 49 years-old, main drug heroin)

Some women interviewed described using the ADM at night to avoid detection.

It's kind of embarrassing. You don’t want to get seen. [What time did you come?] In the middle of the night. You feel like it's not a good thing. (Joby, 27-year-old woman, main drug methamphetamine)

Many participants, of all genders, also expressed concerns about being identified as a PWID by police who sometimes congregated in a hospital car park opposite the ADM outside the hospital emergency department.

There’s cops all over the footpath outside having a smoke or whatever and I’m just off my chops and I’m thinking, “Look, I just don’t want to talk to them today.” (Paul, 41-year-old man, main drug methamphetamine)

During fieldwork, there were many occasions when people were reluctant or unwilling to engage with the researchers when they came to use the ADM. Observations strongly suggested these people were keen to avoid being seen or attracting attention to their activities.

A car pulled into a parking space near the ADM and a woman who looked Southern European (in her 50s) got out and stood in front of the machine, looking unsure of what to do. “Want a hand?” I offered. She did and took a fit-pack. She declined to do a survey saying, “No I really shouldn’t be here”, tucking the fit-pack down the back of her jeans. When she got back in the car, I noticed a teenage girl in the passenger seat. (8/11/2020)

Late at night a car pulled up, very close to the peer worker who was standing next to the machine doing the survey with a participant. The driver seemed at least 65-years-old, short and almost hidden behind the steering wheel. The passenger door opened and a person emerged wearing a long blonde wig, heavy face make-up, a gold dress, black fish-net stockings with suspenders and stilettos. The person walked confidently up to the machine, right next to the participant doing the survey, and got a fit-pack. The person then walked straight back to the car, not engaging with any of us. The car reversed out immediately and off they went. (8/11/2020)

On a minority of occasions, often in the late afternoon, people in suits who were driving expensive cars accessed the ADM but avoided engagement.

A lean man who looked about 60-years-old, with a full head of silver hair, drove up in a shiny black Mercedes. He left his car idling as he quickly used the machine. We noticed his flashy light grey well-cut suit pants and crisp white shirt. He smiled at us on his way past but refused to engage any further. (6/10/2020)

Fieldwork late at night also revealed that the ADM was occasionally accessed by hospital patients.

A man with a partly shaven head and wearing a dressing gown emerged from the main hospital entrance, pushing a drip stand that bumped along beside him. He didn’t engage with us at all. He got one fit-pack, turned around and slowly trudged back into the hospital. (6/10/2020)

At times, the team’s presence may have deterred people from accessing the ADM.

A car with a P plate [provisional driver] drove into the turning circle around 10am, and cruised slowly past the ADM. The driver and passenger both looked male, the latter with his hood up, and there was someone in the back seat. The car stopped some distance away, idled for a few minutes, then drove away. (11/11/2020)

### Lack of time

Many people made a quick approach when using the ADM and some were clearly in a rush.

A man in a well-worn singlet and shorts, maybe in his 40s, was running, barefoot, up to the machine. He looked at us and we said, ‘Hi’, but he didn’t reply. He started pressing buttons on the ADM, the left-hand row only, not knowing how to use it. He asked, “Are there none in there?” I said, “There should be”. I showed him, he took one fit-pack and immediately ran off. (25/9/2020)

Lack of parking meant some people left their car idling near the ADM while they rushed to use it, sometimes blocking the traffic flow.

A BMW drove up, its front bumper partially hanging off. It idled in the turning circle and a woman who appeared to be in her 40s or 50s jumped out and quickly used the machine, taking two fit-packs. The bald man behind the wheel didn’t take his eyes off me as she got the fits. She looked straight at me but blanked me after I said, “Hi”. She got back in the car and it moved off quickly, its exhaust pipe loud. (6/11/2020)

Sometimes lack of time or keeping someone waiting were polite or discrete ways to shut down engagement.

A young guy, probably in his 30s, came to use the machine. He had no idea how to and accepted my offer of help. He declined survey participation saying, “No, I’ve got somebody waiting” and starting walking away. He turned back to say, “And it’s embarrassing”. (22/10/2020)

### Intruding on private spaces

It was crucial that people were free to use the ADM without interacting with the researchers if preferred. However, positioning the team to ensure we were perceived as non-threatening and not hiding from view but also a discrete presence that would not attract attention, proved challenging. The researchers were typically about ten metres from the ADM, in plain sight in a paved area that was illuminated at night, as was the alcove where the ADM was situated. The window of opportunity to engage was narrow. The team waited for eye contact or some acknowledgement of the team’s presence as the person approached the ADM. When a connection was established, the team reassured people we were researchers, drawing their attention to a recruitment flyer on the ADM and that a supermarket voucher was provided for survey participation. Despite this gentle approach, on two or three occasions, people reported feeling “surprised” or “crept-up on”. These observations suggested some people were very focused or anxious at the time they came to use the ADM.

In summary, while the involvement of peer workers and use of incentives appeared to support engagement with people who came to use the ADM, concerns about being seen using the ADM, lack of time and researchers intruding on a private space cast doubt on the acceptability of seeking direct engagement using this approach.

3. *Uptake of hepatitis C testing and treatment*

**Fig. 2:**
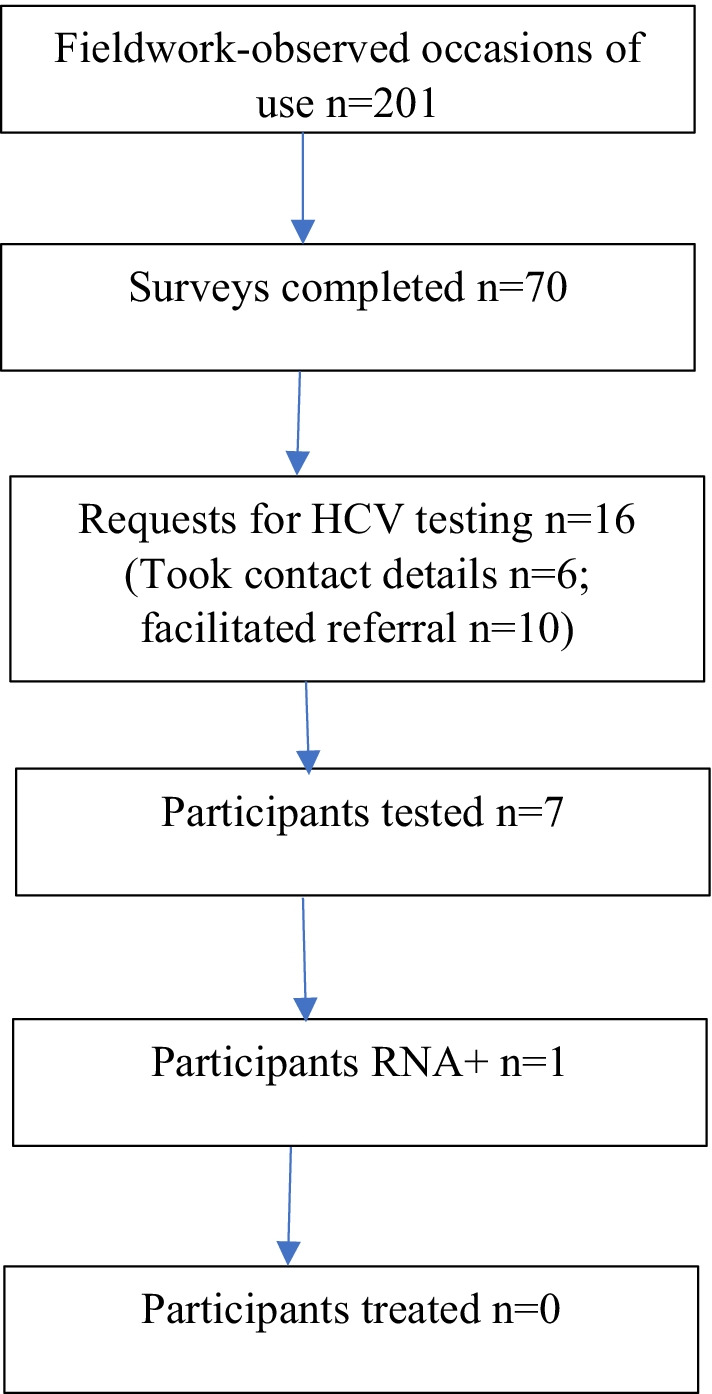
Uptake of HCV testing and treatment

All survey participants (*n* = 70) were offered HCV testing and treatment. Sixteen participants expressed interest in HCV testing, all with the Nurse Practitioner, and seven were tested (Fig. 2). One participant was HCV RNA positive but did not initiate treatment within the study follow-up period.

## Discussion

The study shed light on the characteristics of a population about whom little is known in relation to uptake of HCV testing and DAA treatment, who often choose anonymity and therefore remain hidden by design. Quantitative and qualitative findings highlighted that there is scope for engaging face to face with a diverse population of PWID who may not be engaged with services, at the time they come to use ADMs [7, 20].The involvement of peer workers [27, 28] and the use of financial incentives appeared to be worthwhile strategies for facilitating engagement and survey participation, which warrants further research.

Data suggested the population using ADMs may require HCV treatment and should be considered in HCV elimination efforts. Forty percent of observed occasions of use were by people aged in their 20 s and 30 s, a group identified in previous studies with a high prevalence of syringe sharing [29] but lower use of NSPs compared to older PWID [30]. Among survey participants, 41% reported injecting daily or more, and 39% had not been tested for HCV in the last 12-months. Methamphetamine was the most commonly reported last drug injected for survey participants (58%), indicating that outreach at ADMs provides opportunities for engaging with this group of PWID who may be at risk of HCV infection [31, 32] but may not follow pathways into regular contact with face-to-face services in the way people who are opioid-dependent often do [33].

However, data raised questions about whether seeking to extend the HCV cascade of care to ADMs should be a priority for achieving HCV elimination and whether this approach is consistent with the role of ADMs in harm reduction. How far should services go in their efforts to link people with HCV care? Most people accessing the ADM during fieldwork did not engage with the research team. The survey sample was a minority of those using the ADM so the capacity to draw inferences from these data was limited. However, it was noteworthy that most survey participants reported prior HCV testing, 61% of whom had been tested within in the last 12 months. More than half of survey participants had ever been in OAT. It may be the case that it was people who were unwilling to engage who were the priority group for linkage to HCV care. The study found little evidence to support the view that people who use ADMs could not access HCV testing through other links with health services, if they were interested in doing so. Given the low uptake of HCV testing coupled with the resources required for this type of outreach, it is unlikely to be a cost-effective, sustainable approach, especially in rural or regional areas, where the need for anonymity may be even greater.

Fieldwork also highlighted that extending the HCV cascade of care to ADMs may encroach on what is a private space for some PWID, utilized specifically to avoid engagement. The current study’s findings are an important reminder of the need to ensure public health interventions reflect the needs of affected communities, including their right to remain anonymous. The risk that outreach at ADMs may deter some people from accessing sterile injecting equipment, the single commodity this service provides, could potentially put this approach at odds with harm reduction practices and HCV prevention. When PWID access fixed site, outreach or mobile NSPs, interactions with staff are assumed. The presence of service providers at ADMs may be an unwelcome surprise. In this context, seeking engagement with people who use ADMs may require long-term investment in a range of approaches beyond simply being present in the vicinity of the ADM. Using word-of-mouth and peer workers to gradually engage and build trust with sub-groups of this population over time may be worthy of further investigation.

While the study highlighted that the acceptability of engaging with health workers at the time people come to use ADMs varied, uptake of HCV testing may have been enhanced if was provided “on-the-spot” with DBS or point-of-care testing options [14]. The use of fixed testing times, to forewarn people using the ADM of the presence of service providers or use of a mobile van for discrete HCV testing in the vicinity of the ADM, may enhance acceptability of this approach. Distribution of self-testing kits within networks of people who use ADMs may be preferable.

### Study strengths and limitations

While the study took a novel approach to exploring the effectiveness and acceptability of engaging face-to-face with people at the time they came to use the ADM, there were several methodological limitations of the study, largely related to many people’s reluctance to engage with the researchers beyond a brief exchange. Fieldwork may have deterred some people from accessing the ADM, resulting in underestimating occasions of use and failing to engage with some groups. The gender and age of people observed using the ADM were estimated by the research team rather than self-reported. Errors may have occurred when estimating the number of observed repeat uses of the ADM, due to recall errors and difficulties identifying people wearing head covering. The brief survey was designed for fast administration and could only gather limited information. The survey sample was small and not representative of ADM users. However, the inclusion of fieldnotes and interview data served to strengthen the study findings and assisted in drawing inferences from the data.

### Concluding remarks

ADMs are a highly valued harm reduction service affording maximum anonymity for those seeking to avoid the stigma associated with injecting drug use. Outreach at ADMs provides unique opportunities for engagement with a diverse population who may be unaware they have acquired HCV infection or may not be currently engaged with health services. However, while people who use ADMs may be an important population to target for HCV elimination, questions remain regarding the feasibility and acceptability of extending the HCV cascade of care to a harm reduction service accessed by many people seeking to avoid face-to-face engagement. The research highlighted the complexities of finding the right balance between public health priorities and respecting the privacy of affected communities.

## Data Availability

The datasets generated and/or analysed during the current study are not publicly available due to participant privacy issues, but are available from the corresponding author on reasonable request.

## Abbreviations

ADM: Automatic dispensing machines
ADMiT: ADM users into treatment
CCTV: Closed-circuit television
DAA: Direct-acting antiviral
DBS: Dried blood spot
HCV: Hepatitis C virus
NSP: Needle and syringe programs
OAT: Opioid agonist treatment
PWID: People who injecting drugs
